# Association between the red cell distribution width and mortality in elderly patients with non-traumatic coma: An observational cohort study

**DOI:** 10.1097/MD.0000000000038773

**Published:** 2024-06-28

**Authors:** Dongki Kim, Donghun Lee, Jiho Lee, Byungkook Lee, Sang Won Ko

**Affiliations:** a Department of Emergency Medicine, Chonnam National University Hospital, Gwangju, Republic of Korea; b Department of Emergency Medicine, Chonnam National University Medical School, Gwangju, Republic of Korea.

**Keywords:** age, coma, prognosis, red cell distribution width

## Abstract

Elevated red blood cell distribution width (RDW) can be associated with disease severity. However, studies on RDW for the prognosis of elderly patients with non-traumatic coma (NTC) are lacking. This study aims to examine the relationship between RDW and outcomes in elderly patients with NTC. This observational cohort study included elderly patients (aged ≥ 65 years) with NTC between January 2022 and December 2022. We measured RDW upon patient arrival at the emergency department (ED). We conducted a multivariable analysis using logistic regression of relevant covariates to predict in-hospital mortality. Survival curves based on 30-day mortality were designed using the Kaplan–Meier method. The primary outcome was in-hospital mortality, and the secondary outcome was 30-day mortality. A total of 689 patients were included in the study, and in-hospital mortality was 29.6% (n = 204). Our results found that the RDWs of non-survivors were significantly greater than those of survivors (14.6% vs 13.6%). Multivariable analysis showed that RDWs at ED arrival were independently associated with in-hospital mortality (odds ratio, 1.126; 95% confidence interval, 1.047–1.212; *P* < .001). The Kaplan–Meier curve indicated that the survival probability of patients with a low RDW was greater than those with a high RDW. Having a high RDW at ED arrival was associated with in-hospital mortality in elderly patients with NTC.

## 1. Introduction

Patients with non-traumatic coma (NTC) account for around 1% of emergency department (ED) admissions, adding to the workload of emergency medicine physicians.^[1,2]^ Patients with NTC are a high-risk group, so early diagnosis and treatment are crucial in determining their prognosis.^[3–6]^ The number of elderly patients being admitted to EDs is steadily increasing, with patients over the age of 65 now having the highest percentage of emergency room visits of any age group.^[7]^ Also, because of the complex interactions of both internal and external factors, the mortality in this age group is also higher than in other age groups.^[8]^ Furthermore, mortality risk will increase further in the case of elderly patients with NTC. Therefore, it is important to rapidly identify factors that can determine the prognosis in these patients and to provide prompt and intensive treatment when a poor prognosis is predicted.

Clinical instruments such as the National Early Warning Score, Modified Early Warning Score, Acute Physiology and Chronic Health Assessment, and the Rapid Sequential Organ Failure Score used in critically ill patients can help predict prognosis in elderly patients with NTC.^[9,10]^ However, these tools need to consider objective prognostic predictors because the evaluation processes are often cumbersome and subjective evaluations. Red blood cell distribution width (RDW) is routinely evaluated to gather information about the heterogeneity of circulating red blood cell size as a fraction of the total blood count. The normal erythrocyte size is usually between 80 and 100 fL but varies depending on physiological and pathological conditions.^[11]^ Many studies have shown that RDW can predict the prognosis of various diseases such as heart failure, diabetes mellitus, cerebral vascular disease, out-of-hospital cardiac arrest, colon cancer, and celiac disease.^[12–14]^ In addition, given that RDW is routinely reported as a complete blood count (CBC) component in clinical laboratories and is available to most patients, comprehending the relationship between RDW and patient outcome can be particularly useful for risk stratification in clinical decision-making.

We hypothesize that RDW would be helpful in predicting the death of elderly patients with NTC who visited the ED and aim to examine the relationship between RDW and outcomes in elderly patients with NTC.

## 2. Materials and methods

### 2.1. Study design and population

We conducted this observational cohort study of elderly (aged ≥ 65 years) patients with NTC who visited the ED at Chonnam University Hospital, Gwangju, Republic of Korea between January 2022 and December 2022. NTC is defined as having a Glasgow Coma Scale (GCS) score of ≤ 8.^[15]^ Each patient GCS score was determined by specially trained study nurses and residents. The different coma etiologies were classified into 2 main groups—metabolic coma and structural coma—as described by Plum and Posner.^[6]^ Coma etiologies classified into the structural group were cerebral infarction, intracranial hemorrhage, intracranial tumor, and intracranial infection (meningitis or encephalitis), while the remaining occurring coma etiologies were accordingly classified into the metabolic group and constituted poisoning, epilepsy (status epilepticus, seizures, or postictal state), circulatory failure post-cardiac arrest or circulatory shock, metabolic disorder (hypo/hyperglycemia, hyponatremia, hypothermia, or hepatic failure), and extracranial infection.^[6]^ The exclusion criteria comprised traumatic coma, lack of laboratory measurements, and missing data. Our hospital Institutional Review Board approved the study and informed consent was waived because of the retrospective nature of the study.

### 2.2. Data collection

Data were collected from hospital medical records. Each patient age, sex, etiology of coma, vital signs on ED arrival (systolic blood pressure [SBP, mm Hg], respiratory rate, pulse rate, and body temperature [BT, °C]), initial GCS score at ED arrival, laboratory results on ED arrival (CBC parameters including RDW, blood urea nitrogen [BUN], serum creatinine, and serum electrolytes), and in-hospital mortality were obtained for this study. The RDW results upon arrival at ED were categorized based on quartiles and the median value: <12.9%; 12.9% to 13.9%; 13.9% to 15.5%; and >15.5%. The primary outcome was in-hospital mortality, and the secondary outcome was 30-day mortality.

### 2.3. Statistical analysis

Continuous variables that did not satisfy the normality test are presented as median values with interquartile ranges, while categorical variables are presented as frequencies and percentages. Differences between the 2 groups were assessed using a Mann–Whitney U-test for continuous variables, while Fisher exact test or a chi-squared test was used to compare categorical variables, where appropriate. We also performed a multivariable logistic regression analysis, incorporating relevant covariates, to predict both in-hospital and 30-day mortalities. Variables with *P* values < .20 in the univariable analysis were included in the multivariable regression model. We used a backward stepwise approach and sequentially eliminated variables with *P* values > .10 to build a final adjusted regression model. Finally, for in-hospital mortality (Supplementary Tables 1, http://links.lww.com/MD/N99), the adjusted variables selected were GCS score, systolic blood pressure (SBP), platelet count, and potassium level. For 30-day mortality (Supplementary Tables 2, http://links.lww.com/MD/N100), age, GCS score, SBP, platelet count, and potassium level were selected as adjusted variables. To further improve model accuracy and interpretability, we included age and gender as potential confounding factors in each final model. Subsequently, we included RDW as both continuous and categorical variables in the final model and performed separate analyses. Calibration was assessed using the Hosmer–Lemeshow goodness-of-fit C-statistics, with a *P* > .05 indicating good calibration. We presented logistic regression analysis results as odds ratios (ORs) and 95% confidence intervals (CIs). Survival curves of categorized RDW groups based on 30-day mortality were designed using the Kaplan–Meier method, and comparisons were made using the log-rank test. All analyses were performed using PASW/SPSS™ version 18 (IBM Inc., Chicago, IL) and MedCalc version 19.0 (MedCalc Software Bvba, Ostend, Belgium). A 2-sided significance level of 0.05 denoted statistical significance.

## 3. Results

### 3.1. Patient selection and characteristics

**Figure 1.**
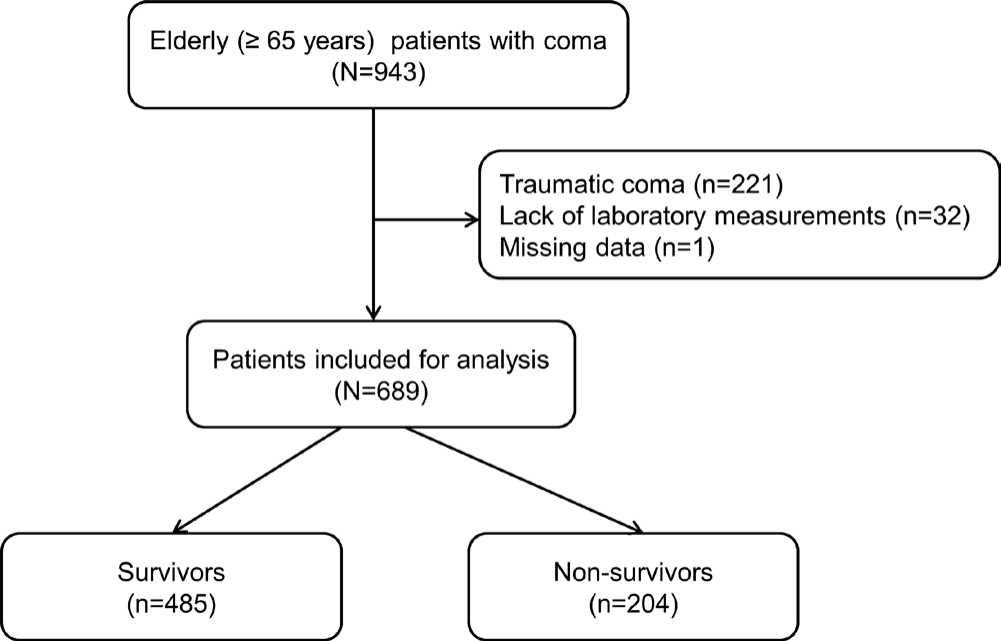
Schematic diagram showing the number of patients included in the present study who were admitted with a comatose level of consciousness.

In total, 943 comatose patients were identified as having met the inclusion criteria during the study period. However, after the exclusion criteria were applied, 689 patients were finally included in this study (Fig. 1). The median age of the patients was 80.0 (73.0–85.0) years, and 353 of the patients were men (51.2%). Among the study population, 534 patients (77.5%) were classified into the metabolic coma group and 155 (22.5%) into the structural coma group. The number of non-survivors was 204 (29.6%). The most common causes of NTC in the metabolic coma group were extracranial infection and cardiac arrest, whereas stroke (cerebral infarction or intracranial hemorrhage) was the most frequent cause in the structural coma group (Table 1).

**Table 1:** The study population (n = 689) divided into cases with metabolic or structural coma and into the different subclasses of coma etiologies.

Metabolic coma	n (%)	Structural coma	n (%)
Poisoning Epilepsy Extracranial infection Cardiac arrest Circulatory shock Respiratory failure Hypoglycemia Hyperglycemia Hyponatremia Hepatic failure Hypothermia	66 (12.4) 36 (6.7) 128 (24.0) 117 (21.9) 76 (14.2) 41 (7.7) 10 (1.9) 10 (1.9) 18 (3.4) 18 (3.4) 14 (2.6)	Cerebral infarction Intracranial hemorrhage Intracranial tumor Intracranial infection	65 (41.9) 69 (44.5) 10 (6.5) 11 (7.1)

### 3.2. Comparison of baseline and clinical characteristics between survivors and non-survivors

Table 2 shows the baseline and clinical characteristics of the survivors and non-survivors. According to hospital data, non-survivors had lower SBP, hemoglobin level, platelet count, and calcium level, and higher RDW, BUN, creatinine, potassium, and chloride levels than survivors.

**Table 2:** Comparison of baseline characteristics of elderly patients with NTC according to in-hospital mortality.

Variables	Total patients (N = 689)	Survivors (N = 485)	Non-survivors (N = 204)	*P* value
Age, yr	80.0 (73.0–85.0)	79.0 (73.0–84.0)	81.0 (75.0–85.0)	.094
Male, n (%)	353 (51.2)	245 (50.5)	108 (52.9)	.618
Cause, n (%)				<.001
Metabolic	534 (77.5)	352 (72.6)	182 (89.2)	
Structural	155 (22.5)	133 (27.4)	22 (10.8)	
GCS score	7 (3–7)	7 (7-7)	6 (3–7)	<.001
SBP, mm Hg	110 (80–140)	120 (100–150)	89 (60–118)	<.001
Respiratory rate,/min	20 (20–22)	20 (20–22)	20 (20–20)	<.001
Pulse rate,/min	91 (76–110)	90 (76–110)	92 (74–116)	.916
Body temperature, °C	36.2 (36.0–37.0)	36.4 (36.0–36.8)	36.1 (36.0–36.7)	.005
Blood cell count				
White blood cell count, ×10^9^/L	11.6 (7.9–16.1)	11.4 (7.9–15.9)	11.7 (7.6–17.4)	.534
Hemoglobin, g/dL	11.6 (9.8–13.3)	11.9 (10.3–13.6)	10.6 (8.8–12.6)	<.001
Neutrophil count, ×10^9^/L	8.6 (5.2–13.3)	8.7 (5.3–13.2)	8.6 (5.1–14.0)	.961
Lymphocyte count, ×10^9^/L	1.4 (0.8–2.4)	1.3 (0.8–2.2)	1.5 (0.7–3.0)	.304
Platelet count, ×10^9^/L	197 (142–260)	212 (156–266)	162 (107–230)	<.001
Neutrophil-to-lymphocyte ratio	6.0 (2.5–13.3)	6.4 (2.7–13.7)	5.5 (2.2–11.7)	.267
RDW, %	13.9 (12.9–15.5)	13.6 (12.8–15.1)	14.6 (13.5–16.4)	<.001
Kidney function				
Blood urea nitrogen, mg/dL	24.5 (17.1–42.9)	22.5 (16.3–36.0)	32.9 (19.7–56.6)	<.001
Creatinine, mg/dL	1.1 (0.8–1.9)	1.0 (0.7–1.5)	1.4 (1.0–2.6)	<.001
Serum electrolytes				
Sodium, mmol/L	139 (136–143)	139 (136–142)	140 (136–144)	.109
Potassium, mmol/L	4.2 (3.7–4.8)	4.1 (3.7–4.6)	4.5 (3.8–5.1)	<.001
Chloride, mmol/L	105 (101–108)	104 (100–108)	106 (101–110)	.003
Calcium, mmol/L	8.6 (8.0–9.2)	8.7 (8.1–9.2)	8.2 (7.5–8.9)	<.001

GCS = Glasgow Coma Scale, NTC = non-traumatic coma, RDW = red cell distribution width, SBP = systolic blood pressure.

Table 3 shows the baseline and clinical characteristics of survivors and non-survivors according to RDW. Patients with higher RDW levels tended to have a higher proportion of metabolic causes, as well as higher BUN and creatinine levels, and lower SBP, hemoglobin levels, platelet counts, and calcium levels. Patients with higher RDW levels were more susceptible to in-hospital mortality.

**Table 3:** Characteristics of the study patients according to RDW.

Variables	RDW, %				*P* value
	<12.9 (n = 162)	12.9–13.9 (n = 172)	13.9–15.5 (n = 183)	>15.5 (n = 172)	
Age, yr	78.0 (72.0–83.0)	80.0 (73.3–85.0)	79.0 (73.0–85.0)	80.0 (75.0–85.0)	.331
Male, n (%)	77 (47.5)	91 (52.9)	99 (54.1)	86 (50.0)	.618
Cause, n (%)					<.001
Metabolic	109 (67.3)	124 (72.1)	151 (82.5)	150 (87.2)	
Structural	53 (32.7)	48 (27.9)	32 (17.5)	22 (12.8)	
GCS score	7 (7-7)	7 (3–7)	7 (3–7)	7 (3–7)	.212
SBP, mm Hg	123 (100–160)	110 (90–140)	110 (80–140)	100 (60–130)	<.001
Respiratory rate,/min	20 (20–22)	20 (20–20)	20 (20–22)	20 (20–22)	.160
Pulse rate,/min	90 (77–108)	90 (73–108)	95 (80–112)	99 (72–120)	.123
Body temperature, °C	36.3 (36.0–36.7)	36.2 (36.0–36.6)	36.4 (36.0–36.8)	36.3 (36.0–36.8)	.228
Blood cell count					
WBC, ×10^9^/L	11.3 (9.0–15.7)	11.9 (7.1–15.9)	11.6 (7.2–15.9)	11.4 (8.0–18.3)	.649
Hemoglobin, g/dL	13.1 (11.2–14.6)	12.4 (11.1–14.2)	11.2 (9.8–13.0)	9.6 (8.0–11.4)	<.001
Neutrophil count, ×10^9^/L	8.3 (5.6–12.8)	8.3 (4.3–12.6)	8.7 (5.0–13.7)	9.1 (5.4–14.8)	.229
Lymphocyte count, ×10^9^/L	1.5 (0.8–3.1)	1.5 (0.9–2.7)	1.1 (0.7–2.1)	1.4 (0.7–2.2)	.006
Platelet count, ×10^9^/L	216 (169–265)	199 (147–249)	181 (128–243)	195 (111–274)	.002
Neutrophil-to-lymphocyte ratio	6.0 (2.2–12.3)	5.0 (1.9–9.8)	7.2 (3.4–15.1)	6.4 (3.2–15.9)	.011
Kidney function					
Blood urea nitrogen	20.2 (14.9–26.8)	22.4 (15.7–34.3)	28.9 (19.9–48.0)	35.3 (18.5–54.0)	<.001
Creatinine, mg/dL	0.9 (0.7–1.3)	1.0 (0.8–1.4)	1.2 (0.8–2.3)	1.4 (0.9–2.7)	<.001
Serum electrolytes					
Sodium, mmol/L	139 (136–142)	139 (136–142)	140 (135–143)	140 (136–144)	.190
Potassium, mmol/L	4.1 (3.7–4.5)	4.1 (3.7–4.8)	4.3 (3.7–4.7)	4.3 (3.7–5.2)	.063
Chloride, mmol/L	104 (100–107)	105 (101–107)	106 (101–110)	105 (101–111)	<.001
Calcium, mmol/L	8.9 (8.2–9.2)	8.7 (8.1–9.3)	8.4 (7.9–9.0)	8.2 (7.7–8.9)	<.001
In-hospital mortality, %	24 (14.8)	48 (27.9)	60 (32.8)	72 (41.9)	<.001

GCS = Glasgow Coma Scale, RDW = red cell distribution width, SBP = systolic blood pressure.

### 3.3. Association between RDW and in-hospital mortality and 30-day mortality

Table 4 shows the results of the multivariable analysis for predicting in-hospital mortality. As a continuous variable, RDW was independently associated with in-hospital mortality after adjusting for confounders (OR, 1.126; 95% CI, 1.047–1.212). As a categorical variable, the RDW groups of 12.9–13.9% (OR, 1.830; 95% CI, 1.014–3.302), 13.9–15.5% (OR, 1.985; 95% CI, 1.115–3.537), and > 15.5% (OR, 2.552; 95% CI, 1.426–4.567) were associated with in-hospital mortality compared to the RDW group of < 12.9%. The Hosmer–Lemeshow goodness-of-fit C-statistics showed good calibration for RDW as continuous and categorical variables (chi-square = 1.680; *P* = .989 and chi-square = 3.689; *P* = .884, respectively).

**Table 4:** Multivariate logistic regression analysis for predicting in-hospital mortality in elderly patients with NTC.

	Adjusted OR (95% CI)	*P* value	Adjusted OR (95% CI)	*P* value
Age, yr	1.021 (0.996–1.047)	.099	1.021 (0.996–1.047)	.096
Male	1.192 (0.818–1.737)	.359	1.163 (0.799–1.694)	.430
GCS score	0.864 (0.780–0.957)	.005	0.864 (0.780–0.956)	.005
SBP, mm Hg	0.985 (0.980–0.990)	<.001	0.986 (0.981–0.991)	<.001
Platelet count, ×10^9^/L	0.995 (0.993–0.997)	<.001	0.995 (0.993–0.997)	<.001
Potassium, mmol/L	1.313 (1.096–1.575)	.003	1.326 (1.107–1.589)	.002
RDW, %	1.126 (1.047–1.212)	<.001		
RDW, <12.9%			Reference	
RDW, 12.9%–13.9%			1.830 (1.014–3.302)	.045
RDW, 13.9%–15.5%			1.985 (1.115–3.537)	.020
RDW, >15.5%			2.552 (1.426–4.567)	.002

Each of the RDW as continuous and categorical variables was included in the final model and analyzed independently.

CI = confidence interval, GCS = Glasgow Coma Scale, NTC = non-traumatic coma, OR = odds ratio, RDW = red cell distribution width, SBP = systolic blood pressure.

**Figure 2.**
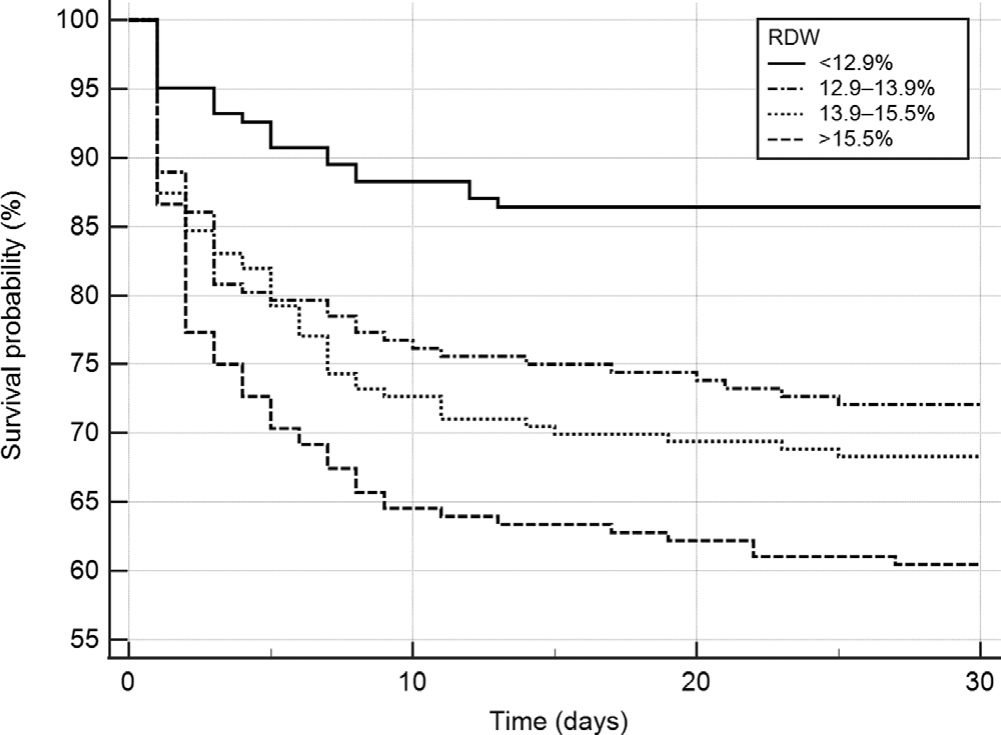
Kaplan–Meier plots for cumulative 30-d survival. The log-rank test showed significant differences (*P* < .001, P-trend < 0.001).

Table 5 shows the results of the multivariable analysis for predicting 30-day mortality. As a continuous variable, RDW was independently associated with in-hospital mortality after adjusting for confounders (OR, 1.127; 95% CI, 1.047–1.213). As a categorical variable, the RDW groups of 12.9–13.9% (OR, 2.035; 95% CI, 1.111–3.726), 13.9–15.5% (OR, 2.086; 95% CI, 1.152–3.779), and > 15.5% (OR, 2.472; 95% CI, 1.357–4.500) were associated with in-hospital mortality compared to the RDW group of < 12.9%. The Hosmer–Lemeshow goodness-of-fit C-statistics showed good calibration for RDW as continuous and categorical variables (chi-square = 4.637; *P* = .796 and chi-square = 9.390; *P* = .310, respectively). Additionally, Kaplan–Meier survival curves for the 4 groups during the 30-day mortality were constructed (Fig. 2) and were shown to be statistically different according to the log-rank test (*P* < .001, P-trend < 0.001).

**Table 5:** Multivariate logistic regression analysis for predicting 30-d mortality in elderly patients with NTC.

	Adjusted OR (95% CI)	*P* value	Adjusted OR (95% CI)	*P* value
Age, yr	1.028 (1.002–1.054)	.036	1.028 (1.002–1.054)	.034
Male	1.106 (0.756–1.619)	.604	1.075 (0.735–1.572)	.710
GCS score	0.873 (0.787–0.968)	.010	0.872 (0.787–0.967)	.010
SBP, mm Hg	0.985 (0.980–0.990)	<.001	0.985 (0.980–0.990)	<.001
Platelet count, ×10^9^/L	0.996 (0.993–0.998)	<.001	0.996 (0.994–0.998)	<.001
Potassium, mmol/L	1.373 (1.142–1.651)	<.001	1.389 (1.156–1.670)	<.001
RDW, %	1.127 (1.047–1.213)	<.001		
RDW, <12.9%			Reference	
RDW, 12.9%–13.9%			2.035 (1.111–3.726)	.021
RDW, 13.9%–15.5%			2.086 (1.152–3.779)	.015
RDW, >15.5%			2.472 (1.357–4.500)	.003

Each of the RDW as continuous and categorical variables was included in the final model and analyzed independently.

CI = confidence interval, GCS = Glasgow Coma Scale, NTC = non-traumatic coma, OR = odds ratio, RDW = red cell distribution width, SBP = systolic blood pressure.

## 4. Discussion

This observational cohort study found that RDWs in elderly patients with NTC were higher in non-survivors than in survivors. Furthermore, RDW was found to be a surrogate marker associated with in-hospital mortality in elderly patients with NTC at ED arrival.

Clinical conditions in which RBCs routinely increase or decrease are usually caused by iron, vitamin B12, and folate deficiency, increased RBC depletion, and inefficient RBC production including blood transfusions.^[16]^ Alterations in erythropoiesis rates can lead to extensive heterogeneity in RBC size, suggesting that pathological changes are occurring in the organism. This leads to changes, and eventually abnormalities, in RDW, which can be associated with the severity of various diseases.^[17]^ Inflammatory cytokines are present in patients with severe diseases with one study showing that elevated RDW is associated with elevated IL-6 levels.^[18]^ Inflammatory cytokines (tumor necrosis factor-a, IL-1b, and IL-6) affect the production of erythropoietin and iron metabolism.^[18]^ Oxidative damage and inflammatory cytokines shorten the lifespan of red blood cells.^[18]^ By this mechanism, RDW increases because patients with severe disease are affected by inflammatory cytokines at various stages of red blood cell production and destruction.^[18]^

The most common causes of metabolic coma in the present study were extracranial infection and cardiac arrest. Considering that the mechanism leading to death in these 2 diseases is systemic inflammation and subsequent multiple organ failure, it is not surprising that RDW is associated with in-hospital mortality.^[19,20]^ Regarding stroke, which is the most common cause of structural coma in the present study, most causes of in-hospital death were heart disease, pneumonia, or cancer, this can also be related to RDW.^[21]^ In addition, patients with high RDW are more likely to have higher BUN and creatinine levels, and lower hemoglobin levels and platelet counts.

Our findings suggest that the overall health of patients with high RDW levels was worse than that of patients with low RDW levels. In particular, it is well known that BUN, creatinine and hemoglobin levels, and platelet counts are significantly associated with prognosis in elderly patients.^[22–25]^

A previous study showed similar results to the present study. In the previous study, patients over 50 years of age who visited the emergency room in a coma were classified into 2 groups: a metabolic disorder group and a structural disorder group, and the respective rates were 72% and 28%.^[5]^ However, in the present study, one of the most common causes of metabolic coma was cardiac arrest. This may be because the hospital where our study was conducted is a tertiary medical institution, and many patients are referred there for post-resuscitation care after cardiac arrest.

One study also showed a relationship between platelets and 28-day mortality in patients with sepsis.^[26]^ In this study, mortality increased as platelets decreased in patients with a Simplified Acute Physiology Score II score of 65 or higher.^[26]^ In addition to their important role in hemostasis, platelets play an important role in inflammatory diseases. Another study showed that platelets have immunogenic capabilities.^[27]^ Like traditional innate immune cells, platelets are aggregated into injured tissues where they release immune mediators and express immune-active membrane receptors, which interact with other immune cells to identify and eliminate pathogens.^[27]^ In addition, another study showed that the in-hospital mortality rate increased as the RDW to platelet ratio increased in critically ill patients with acute kidney injuries, which was in line with the results obtained in our study.^[28]^

Several studies showed that the ion shift index helps predict prognosis in illnesses involving systemic inflammation, such as severe trauma or cardiac arrest.^[29,30]^ This is in line with the association between changes in serum electrolytes and prognosis in illnesses involving severe inflammatory responses.^[31]^ The ion shift index includes potassium levels.^[29,30]^ In an observational study involving 98 patients with cardiac arrest, high potassium levels were associated with high mortality.^[31]^ A large retrospective observational study of critically ill patients admitted to an intensive care unit showed that a potassium level of > 4.5 mEq/L at admission was a strong independent predictor of 30-day mortality.^[32]^

The present study had several limitations. First, it was a retrospective study performed at a single center; thus, its findings are not immediately generalizable to the overall population. Second, the various hemodynamic factors were measured and evaluated by different people, leading to variability. The respiratory rate in particular had limitations in measurement, so it was more difficult to reflect it in the results. Third, a comprehensive assessment of comorbidity burden was not possible as such data is not the part of Charson Cormorbility Index elements, which most intensive care unit admission registries are based on. Finally, 33 patients were excluded from the present study: blood sampling was not performed in 32 patients, and accurate GCS was not measured in 1 patient. Selection bias may occur due to excluded patients, but the proportion (33/722 = 4.6%) was low and may not significantly affect the main results.

## 5. Conclusion

An elevated RDW is independently associated with in-hospital mortality in elderly patients with NTC. The association was still significant after adjustment for hospital risk factors and important laboratory variables.

## Author contributions

**Conceptualization:** Donghun Lee.

**Data curation:** Dongki Kim, Donghun Lee.

**Formal analysis:** Dongki Kim, Donghun Lee.

**Funding acquisition:** Dongki Kim.

**Investigation:** Dongki Kim, Donghun Lee, Jiho Lee.

**Methodology:** Donghun Lee, Byungkook Lee.

**Project administration:** Donghun Lee.

**Resources:** Donghun Lee.

**Software:** Donghun Lee.

**Supervision:** Byungkook Lee, Sang Won Ko.

**Writing – original draft:** Dongki Kim, Donghun Lee, Jiho Lee.

**Writing – review & editing:** Dongki Kim, Donghun Lee, Jiho Lee, Sang Won Ko.

## Supplementary Material




