# Supplementary material for: Association between the red cell distribution width and mortality in elderly patients with non-traumatic coma: An observational cohort study
Source: Medicine (Baltimore). 2024 Jun 28;103(26):e38773. doi: 10.1097/MD.0000000000038773 (PMC11466147; doi:10.1097/MD.0000000000038773)
Supplement: Supplementary file 1 [file medi-103-e38773-s001.docx]

Supplementary Table 1. Multivariate logistic regression analysis for predicting in-hospital mortality in elderly patients with NTC

|  | Adjusted OR (95% CI) | P-value |
| --- | --- | --- |
| Age, years | 1.021 (0.996–1.047) | 0.094 |
| Metabolic cause | 1.448 (0.829–2.528) | 0.193 |
| GCS score | 0.880 (0.794–0.975) | 0.014 |
| SBP, mmHg | 0.986 (0.981–0.991) | <0.001 |
| Respiratory rate, /min | 0.971 (0.933–1.012) | 0.161 |
| Body temperature, ℃ | 0.941 (0.780–1.133) | 0.520 |
| Hemoglobin, g/dL | 0.983 (0.907–1.065) | 0.668 |
| Platelet count, ×10^9^/L | 0.995 (0.993–0.997) | <0.001 |
| Blood urea nitrogen, mg/dL | 1.006 (0.999–1.012) | 0.102 |
| Creatinine, mg/dL | 1.010 (0.891–1.146) | 0.875 |
| Sodium, mmol/L | 1.006 (0.981–1.030) | 0.654 |
| Potassium, mmol/L | 1.389 (1.160–1.664) | <0.001 |
| Chloride, mmol/L | 0.989 (0.944–1.037) | 0.655 |
| Calcium, mmol/L | 0.815 (0.664–1.000) | 0.050 |

NTC, non-traumatic coma; OR, odds ratio; CI, confidence interval; GCS, Glasgow Coma Scale; SBP, systolic blood pressure; RDW, red cell distribution width
