# Supplementary material for: Association between the red cell distribution width and mortality in elderly patients with non-traumatic coma: An observational cohort study
Source: Medicine (Baltimore). 2024 Jun 28;103(26):e38773. doi: 10.1097/MD.0000000000038773 (PMC11466147; doi:10.1097/MD.0000000000038773)
Supplement: Supplementary file 2 [file medi-103-e38773-s002.docx]

Supplementary Table 2. Multivariate logistic regression analysis for predicting 30-day mortality in elderly patients with NTC

|  | Adjusted OR (95% CI) | P-value |
| --- | --- | --- |
| Age, years | 1.028 (1.002–1.054) | 0.035 |
| Metabolic cause | 1.370 (0.776–2.419) | 0.278 |
| GCS score | 0.889 (0.801–0.986) | 0.026 |
| SBP, mmHg | 0.985 (0.980–0.990) | <0.001 |
| Respiratory rate, /min | 0.971 (0.933–1.012) | 0.162 |
| Body temperature, ℃ | 0.902 (0.747–1.090) | 0.286 |
| Hemoglobin, g/dL | 1.013 (0.934–1.099) | 0.755 |
| Platelet count, ×10^9^/L | 0.996 (0.994–0.998) | <0.001 |
| Blood urea nitrogen, mg/dL | 1.005 (0.999–1.012) | 0.113 |
| Creatinine, mg/dL | 0.995 (0.875–1.132) | 0.942 |
| Sodium, mmol/L | 1.003 (0.979–1.028) | 0.817 |
| Potassium, mmol/L | 1.453 (1.209–1.054) | 0.035 |
| Chloride, mmol/L | 0.989 (0.943–1.037) | 0.648 |
| Calcium, mmol/L | 0.811 (0.659–1.000) | 0.051 |

NTC, non-traumatic coma; OR, odds ratio; CI, confidence interval; GCS, Glasgow Coma Scale; BP, systolic blood pressure; RDW, red cell distribution width
